# Prelingual Sensorineural Hearing Loss Caused by a Novel *GJB2* Dominant Mutation in a Chinese Family

**DOI:** 10.1155/2020/6370386

**Published:** 2020-01-21

**Authors:** Shasha Huang, Xue Gao, Yufeng Wang, Dongyang Kang, Xin Zhang, Suyan Yang, Pu Dai

**Affiliations:** ^1^Department of Otolaryngology, PLA General Hospital, Do. 28 Fuxing Road, Beijing 100853, China; ^2^Department of Otolaryngology, PLA Rocket Force Characteristic Medical Center, 16# XinWai Da Jie, Beijing 100088, China; ^3^Department of Hospitalization Management, PLA General Hospital, Do. 28 Fuxing Road, Beijing 100853, China

## Abstract

**Background:**

*GJB2* mutation is the most common cause of genetic deafness. Many pathogenic variations have already been identified, and thus, fewer and fewer novel pathogenic variations remain to be identified. Here, we describe a novel pathogenic variation associated with dominant hereditary deafness in a Chinese family.

**Methods:**

In this study, we examined four generations of a Chinese family (M127) with hearing loss. Temporal CT scan, complete physical examination (including skin and hair), and audiological tests were performed. Targeted next-generation and Sanger sequencing were used to identify pathogenic mutations in affected individuals.

**Results:**

All patients exhibited prelingual nonsyndromic sensorineural hearing loss, with severity ranging from moderate to severe. A novel dominant pathogenic variant c.205T > C (p.Phe69Leu) was identified in all patients in this family.

**Conclusions:**

c.205T > C (p.Phe69Leu) was identified as a novel dominant pathogenic variant of *GJB2* associated with prelingual nonsyndromic sensorineural hearing loss.*GJB2* mutation is the most common cause of genetic deafness. Many pathogenic variations have already been identified, and thus, fewer and fewer novel pathogenic variations remain to be identified. Here, we describe a novel pathogenic variation associated with dominant hereditary deafness in a Chinese family.

## 1. Introduction

Pathogenic mutation of *GJB2*, which encodes the gap junction protein connexin 26 (Cx26), is the most common cause of hearing loss in many populations. Pathogenic variants of *GJB2* have been linked to both autosomal recessive hereditary sensorineural hearing loss (DFNB1) and autosomal dominant hereditary sensorineural hearing loss (DFNA3). Currently, more than 200 pathogenic variants have been identified, including both recessive and dominant mutations, related to nonsyndromic or syndromic deafness. For recessive pathogenic variants, differences in hotspot variants have been shown to be a characteristic of different nationalities. In contrast, there are currently 40 dominant pathogenic variants related to hearing loss or syndromic hearing loss [1–3], with no evidence of racial differences. Here, we describe a novel dominant pathogenic variant of *GJB2* (c.205 T > C; p.Phe69Leu) associated with prelingual nonsyndromic sensorineural hearing loss in a Chinese family.

## 2. Materials and Methods

### 2.1. Clinical Data

Individuals representing four generations of a single Chinese family (Family M217) were examined. A total of 16 family members, including 6 clinically affected with hearing loss and 10 unaffected members, were recruited in this study. Clinical information was gathered through multiple interviews with all participating members of the family. Medical history, otoscopy, physical examination, and pure tone audiometric examinations were performed for all patients. Computed tomography (CT) scans of the temporal bones of proband and his father were performed.

### 2.2. Mutational Detection and Analysis

#### 2.2.1. Targeted Deafness Gene Capture and Next-Generation Sequencing (NGS)

Informed written consent was obtained from all subjects or their guardians for genetic analysis and publication. The study was approved by the Ethics Committee of Chinese PLA General Hospital. Genomic DNA was extracted from peripheral blood using a blood DNA extraction kit according to the manufacturer's instructions (Qiagen, Hilden, Germany).

The proband was tested with a panel containing 133 deafness genes, 6 mitochondrial regions associated with deafness, and 3 deafness-related microRNAs (Supplemental [Supplementary-material supplementary-material-1]). All oding exons, along with 100-bp flanking regions, were captured for each of the 133 genes and sequenced. Details of the deafness gene capture, sequencing, and bioinformatics analysis methods have been described previously [4].

Pathogenic or likely pathogenic variants detected by NGS in the probands were subsequently verified by polymerase chain reaction (PCR) amplification and Sanger sequencing.

#### 2.2.2. Amino Acid Conservation

Amino acid conservation was evaluated using the HomoloGene database with the sequences of *Homo sapiens*, *Mus musculus*, *Gorilla gorilla*, *Bos taurus*, *Rattus norvegicus*, and *Xenopus laevis*.

#### 2.2.3. Structure-Based Analysis and Three-Dimensional Model Prediction

Amino acid changes within different protein domains were examined using the National Center for Biotechnology Information (NCBI) Conserved Domain Database. Three-dimensional models of human wild-type and mutant sequences were constructed with the program SWISS-MODEL (http://swissmodel.expasy.org).

## 3. Results

### 3.1. Clinical Evaluations

**Figure 1:**
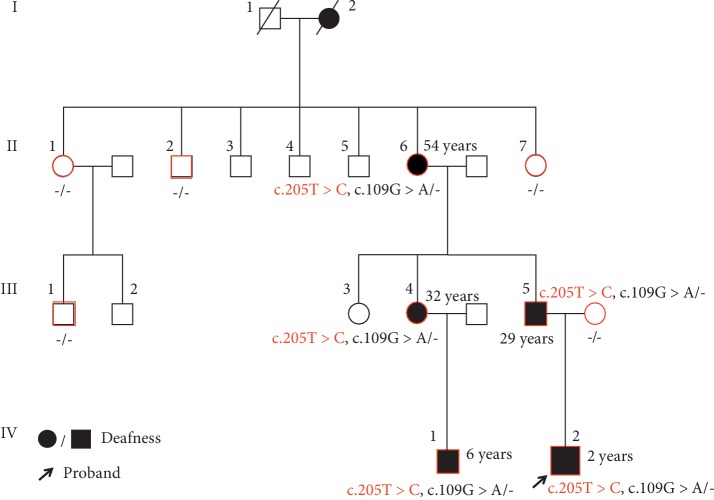
Pedigree of family M217. Family members participating in this study are indicated in red, with *GJB2* gene testing results listed below.

A family tree of family M217 is shown in Figure 1. All family members participating in this study are indicated in red.

**Figure 2:**
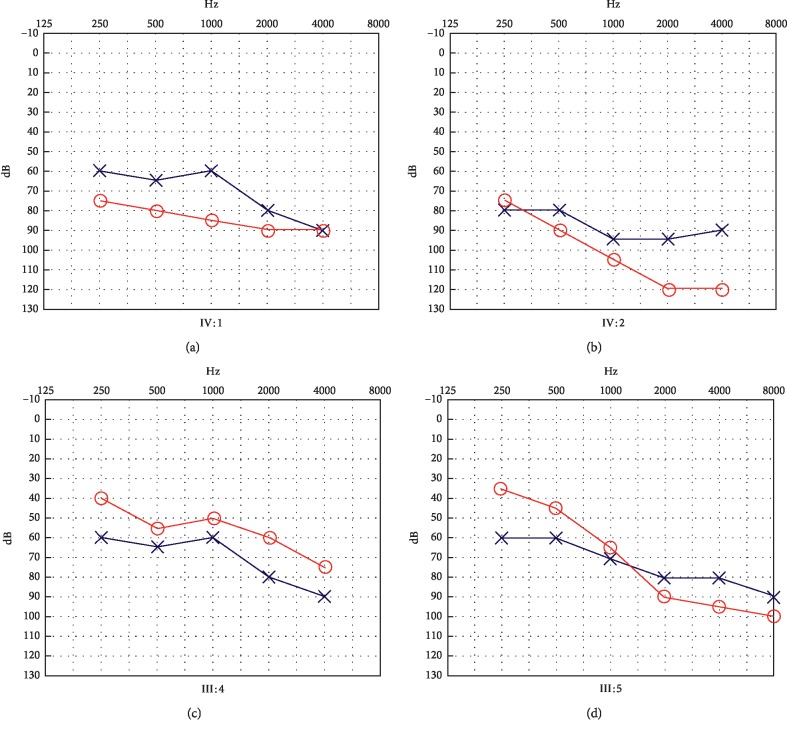
Audiograms of parts of affected members in Family M217: (a) -IV : 1; (b) -IV : 2; (c) -III : 4; (d) -III : 5.

Figure 2 shows the hearing assessments for four patients (IV : 1, IV : 2, III : 4, and III : 5), all of whom were diagnosed with bilateral sensorineural deafness beginning at birth. Bilateral hearing ability was basically symmetrical, with hearing loss curves exhibiting the characteristic slow-drop type (from moderate to severe).

One patient (II : 6) did not undergo a hearing test for this study although her son (III : 5) reported that the patient had hearing loss from childhood. In the absence of direct physical examination, medical records were investigated, revealing that a hearing test had been performed for this patient 10 years prior, demonstrating sensorineural hearing loss in the range of 60–70 dB nHL. Although the patient had been able to communicate normally with the use of hearing aids, her hearing ability gradually decreased over time, and hearing aids were no longer effective. Patient I : 2 also reported hearing abnormalities from childhood and was unable to communicate normally although no hearing test was performed. Based on these findings, it was concluded that all six affected members experienced prelingual nonsyndromic sensorineural hearing loss, with the degree of hearing loss ranging from moderate to severe.

No obvious abnormalities were observed in temporal bone CT of patients III : 5 and IV : 2. No skin phenotypes or other systemic abnormalities were identified through physical examinations.

### 3.2. Molecular Analysis

**Figure 3:**
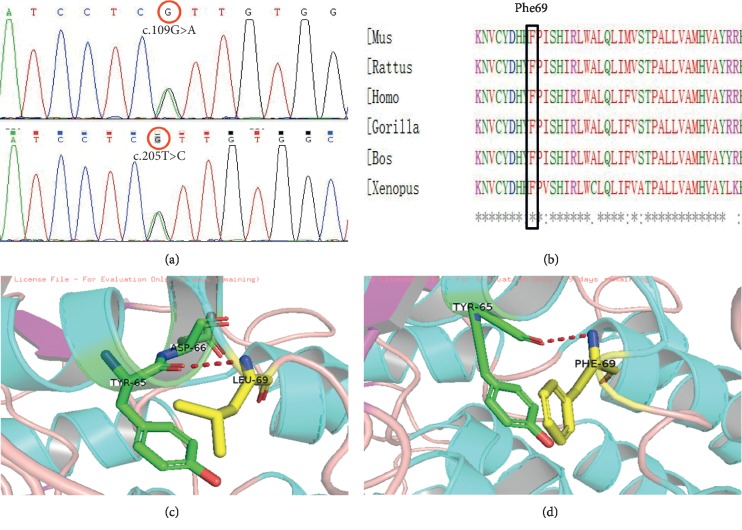
Pathogenic mutation c.205T > C(p.Phe69Leu): (a) chromatograms of c.205 T > C; (b) comparison of amino acid sequences of p.Phe69 among diverse species, showing highly conserved sequences; (c) molecular modeling of wild-type *GJB2*; (d) molecular modeling of wild-type and mutant *GJB2*.

After identification of variants (Supplemental [Supplementary-material supplementary-material-1]), we focused on splice acceptor and donor site mutations and frameshift coding indels, which were more likely to be pathogenic than others (Supplemental [Supplementary-material supplementary-material-1]). According to the pedigree, the inheritance mode of deafness in this family should be autosomal dominant inheritance. So the suspected pathogenic genes were EYA1, COL4A4, and *GJB2*. These variations were sequenced and verified by family members. Finally, two variations were identified in *GJB2*. The first variation, c.205T > C, conferred a phenylalanine to leucine substitution (p.Phe69Leu), whereas the second variation, c.109G > A resulted in a valine to isoleucine substitution (p.Val37Ile) (Figure 3(a)). The two variations were first verified in the parents of affected children. The father (III : 5) was found to be a carrier of both mutations, whereas no variations were detected in the mother (III : 5). These results suggested that both variations were from the father and were located on the same chromosome.

Based on these findings, *GJB2* variant analysis was performed in seven other family members, including three affected and four unaffected individuals, using Sanger sequencing (Figure 1). All affected family members (II : 6, III : 4, and IV : 1) were found to possess both the c.205T > C and c.109G > A variants, with no variants evident in unaffected members (II : 1, II : 2, II : 7, and III : 1). Considering the characteristics of this family and Mendelian heredity, we inferred that patient I : 2 was the source of both the c.205T > C and c.109G > A variants.

### 3.3. Pathogenic Analysis of Variants

The c.109G > A substitution is a well-known autosomal recessive pathogenic variation resulting in significant differences in hearing phenotypes [5, 6]. The amino acid substitution caused by the c.205T > C mutation is located in the first extracellular loop region (EC1) of connexin 26. This mutation has not been reported previously, and is not present in the ExAC database (http://exac.broadinstitute.org/), nor was it detected in the 600 individuals with normal hearing included in this study (Supplemental [Supplementary-material supplementary-material-1]). Furthermore, the substitution occurred in an evolutionarily conserved region of *GLB2* (Figure 3(b)) and was predicted to be damaging by SIFT, Polyphen2, and Mutation Taster. Three-dimensional structural analysis revealed that the p.Leu69 mutation was likely to perturb an amino acid chain (Figures 3(c) and 3(d)). The patient's phenotype and the detected variants have been submitted to the ClinVar (https://www.ncbi.nlm.nih.gov/clinvar/) (Supplemental [Supplementary-material supplementary-material-1]).

## 4. Discussion

Using a combination of pedigree mapping and genetic sequencing, we found that the hearing loss experienced by members of family M217 appears to be the result of autosomal dominant hereditary deafness. We identified a novel variation, c.205T > C (p.Phe69Leu), in *GJB2* in this family. Based on these results, the phenotypes of the family, and the American College of Medical Genetics and Genomics standards and guidelines, the gene variant identified in this study is pathogenic according to the standards of PS4, PM1, PM2, PP1, and PP3 [7]. Within family M217, all affected members were found to possess the c.205T > C and c.109G > A variations. These mutations were consistently passed down as a pair due to their location on the same chromosome, as evidenced by analysis of the individual probands.

It has been controversial about the relationship between c.109G > A variation and the hearing phenotype, but most viewpoints supported c.109G > A was a pathogenic variation. In the case of homozygous or compound heterozygous variations (c.109G > A and the other variations are located on different chromosomes), it was associated with a broad spectrum of hearing phenotypes, ranging from severe-to-profound hearing loss to normal hearing and from congenital onset to delayed onset [8–10]. In this article, c.109G > A and c.205T > C were located on the same chromosome. As the c.109G > A mutation is an autosomal recessive genetic variation, it is unlikely that this variant significantly contributes to the deafness of affected family members.

These data, together with the clinical presentation of the affected siblings and consistent autosomal dominant inheritance of the mutations in the affected and unaffected members, indicate that *GJB2* c.205T > C (p.Phe69Leu) causes hearing impairment in this family. This study represents the first report of this dominant pathogenic variation in *GJB2*.

**Figure 4:**
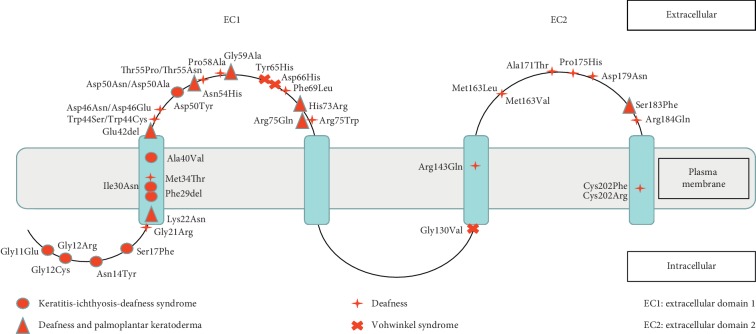
Overview of *GJB2* dominant mutations identified (p.Phe69Leu reported in this study).

Including the variation reported in this study, a total of 41 dominant pathogenic variations have been identified to date. Among these variations, 20 mutations are specific for deafness, whereas the remaining 21 are associated with deafness as well as other symptoms. This includes 10 variations associated with keratitis-ichthyosis-deafness syndrome, three variations associated with Vohwinkel syndrome, and eight variations associated with deafness and palmoplantar keratoderma (Figure 4). However, despite the causative nature of these mutations, there are significant phenotypic differences among patients with the same variation, with deafness occurring at different times and with varying degrees of hearing loss, as well as deafness without other symptoms [11–13].

In this study, patients harboring the c.205T > C variation showed prelingual deafness without other abnormalities, with a progressive decline in hearing ability over time. However, given the variability seen with other mutations, as more and more patients with the c.205T > C variation are found, phenotypic variations may also be found, such as in patients with postlingual deafness or other systemic abnormalities. Long-term follow-up of family M217 will be necessary to monitor the effects of this novel mutation on hearing loss.

## 5. Conclusion

c.205T > C is a novel dominant nonsyndromic pathogenic variation associated with prelingual deafness. More cases are needed to determine whether other abnormalities may be caused by this mutation.

## Data Availability

The data used to support the findings of this study are included within the article.

## Supplementary Materials

Supplementary Materials S1. List of genes tested. S2. Variations of NGS. S3. Likely pathogenic variations. S4. Variations in GJB2 of normal individuals. S5. The patient's phenotype and the detected variants submitted to the ClinVar.
